# Ecological caring—Revisiting the original ideas of caring science

**DOI:** 10.3402/qhw.v11.33344

**Published:** 2016-11-30

**Authors:** Helena Dahlberg, Albertine Ranheim, Karin Dahlberg

**Affiliations:** 1 Institute of Health and Care Sciences, University of Gothenburg, Gothenburg, Sweden; 2 Sektionen för omvårdnad, Institute for neurobiology, care science and society, Karolinska Institutet, Solna, Sweden; 3 Previously at Institute of Caring Sciences and Social Work, Växjö University (now Linnaeus University), Växjö, Sweden

**Keywords:** Ecological care, ecology and health care, phenomenology, Merleau-Ponty, lifeworld-led care

## Abstract

The aim of this empirically grounded philosophical paper is to explore the notion of holistic care with the intention to expand it into a notion of ecological care and in such a way revisit the original ideas of caring science. The philosophical analysis, driven by lifeworld theory and especially Merleau-Ponty's philosophy, is firmly rooted in contemporary clinical care. We used interview data from patients in a study at an anthroposophic clinic in Sweden, which forms part of an ecological community with, for example, ecological agriculture. The empirical study is analysed according to reflective lifeworld research. Starting from the fact that illness can be defined as a loss of homelikeness in the body and in the familiar world, our findings illustrate how ecological care helps the patient to once again find one's place in a world that is characterized by interconnectedness. The task of ecological care is thus not only to see the patient within a world of relationships but to help the patient *find his/her place again, to understand himself/herself and the world anew*. Ecological care is not only about fighting an illness, but also recognizes a patient from inside a world that s/he is affected by and affects, that s/he is understood and understands from. Such care tries to restore this connection by making possible the rhythmical movement as well as the space in-between activity and rest, between being cared for and actively involving oneself in one's recovery and between closing oneself off from the world and once again going out into it.

When the caring professionals’ knowledge base shifted to an academic aim, a sound indication emerged to develop an alternative to medicine's view of humans as the sum of parts, where body and soul are opposites in a strong dualism. In Sweden during the 1980s, there was a fresh nursing education culture with an explicit value basis in human science. Partly inspired by US scholars, nursing students felt compelled to develop what was called holistic nursing, with its core founded in a holistic view of humans (c.f. Drew & Dahlberg, 1995; Eriksson, 2002; Watson, 2008). The holistic view was, in short, an approach with the aim of combining the two aspects of body and soul—occasionally adding a third aspect, the spirit, to be viewed together as a whole person, and not only a patient or a diagnosis. At the same time in Europe, as well as in the United States, a New Age movement emerged with a quasi-scientific approach grounded in religion. Consequently, the nursing and caring science knowledge base attracted the same kind of critique, as the New Age movement, namely, being vague, ill-defined and even “woolly.” In short, the caring science holistic view was considered unscientific.

According to the American Holistic Nurses Association (1998), the concept of holistic care can be defined as a “specialty practice that draws on nursing knowledge, theories, expertise and intuition to guide nurses in becoming therapeutic partners with people in their care. This practice recognizes the interconnectedness of body, mind, emotion, spirit, social/cultural, relationship, context, and environment” (American Holistic Nurses Association, 1998). The definition is vast and rather abstract, posing questions about how holistic care does feature in practice as well as the significant meaning of, for example, environmental milieu. In a letter to the editor of *Journal of Holistic Nursing*, Roller (1995) wrote: “Every article in the journal referred to holism in reductionist terms. In nursing we speak about holism but we constantly refer holistically to the individual as mind-body; or mind, body, and spirit; or a bio psychosocial being. Why do we use reductionist terms to define holism?” A reply unfortunately conveyed that “there is utility in speaking of parts; in so doing, we get a fuller, richer view of the whole.”

A concept analysis of “holistic practice” established that “nursing care embraces the mind, body and spirit of the person, within a culture that supports a therapeutic nurse-patient relationship, which contributes to wholeness, harmony and healing” (McEvoy & Duffy, 2008). They also stated that holistic care “is patient led and patient focused in order to provide individualised care, thereby, caring for the patient as a whole person rather than in fragmented parts” (p. 418). Although we agree with the emphasis made on the relationship between patient centeredness and holistic care, questions once more arise about what holistic care means and how it could or should be incorporated into clinical practice. We are vigilant towards the possibility that “holistic care” often is polarized against a natural scientific view and therefore instigates further nurturance to a dualistic view in health care.

One approach that unites the view of care based in human science and existence with a natural science and biologically based care is the one with its roots in phenomenology and lifeworld theory (cf. Dahlberg & Segesten, 2010; Dahlberg et al., 2008; Dahlberg et al., 2009; Ranheim, 2011; Todres, Galvin & Dahlberg 2007, 2014). Often within caring science theories, health instead of illness is on the foreground with the goal of support and empowerment for the patient's health regime. Subsequently, another goal is well-being, in order for the patient to function optimally in activities of daily living, thus being empowered to cope with different degrees of daily challenges (Ibid.). However, even if a holistic view is present, it is implicit. The tides of changes have arrived, and it has become necessary to revisit the ideas of holistic nursing and caring science in order to determine a bearing significance for the twenty-first century.

## Aim and method

We aimed to explore whether the basic ideas of “holistic caring” can be viewed in innovative ways. In the light of contemporary claims on health care and in order to support further development of health-oriented caring science and nursing, the aim is to explore the meaning of ecological care.

In this philosophical paper, our approach is from within phenomenology (Dahlberg et al., 2008). Explicitly, we wanted to explore how a phenomenological lifeworld theory could help to expand the idea of holistic caring into one that could be called “ecological care”. In particular, our study is founded in Merleau-Ponty's philosophy and especially his understanding of corporeal being (Merleau-Ponty, 1995/1945; Dahlberg, 2013). This philosophy's view is how human beings need to be understood, firstly from the bodily engagement with the cultural and natural world and then with other people. Human existence cannot be defined definitely but defines itself continuously through this engagement. With this philosophy, we can understand how humans take part in, and form their part of the world, and how this knowledge fits within a health care context.

However, the philosophical analysis needs to be evidently rooted in contemporary clinical care, and therefore, as an empirical context for the philosophical analysis, we have used interview data from patients in an investigation at an anthroposophic clinic in Sweden. The care at that clinic is not explicitly described as “ecological care,” but it forms part of an ecological community, which incorporates a health care clinic, ecological agriculture and dairy. Their approach to pedagogy also has relevance since it seeks to develop the complex human being through an integration of theory, arts and practical work, which intends to nourish the individual human being (Dahlin, 2013).

In the following section, we introduce the notion of ecology. Anthroposophic care is described as well as the understanding of such care as ecological. The empirical study, as well as the analysis of data, is also described. The empirical findings are presented at the end of the section. The philosophical analysis is an excursive interpretation of the meanings of ecological care that emerged from the empirical analysis.

## The notion of ecology

Ecology was described by Naess (1976) as the science of living creatures’ relation to and their interaction with the environment, that is, the milieu that we live in and share, a definition that still is valid. In ecological studies, life processes and the development of ecological systems are understood starting from the interdependent relationships between humans and different organisms and their environment. From a human science perspective, ecology means an emphasis on the existential web of relationships, and as such, it may have a significant impact on the understanding of nursing and caring science. It may also be a concept with which we can develop the understanding of what holistic care means.

The concept of ecology in relation to nursing care has previously been elucidated by Gadow (1992). She used the term existential ecology in her argument to reconcile human objectives and natural methods. Her aim was to emphasize a viewpoint, in which nature is experienced as a form of embodied home for human existence. Prior to this argument, the concepts of environment, ecosystems and ecology were applied by Nightingale (1992/1959) and later by Fawcett (1984). According to Lausten (2006), the essential meaning of a nursing ecological theory may guide the profession to new directions of care, striving for the greater good of the patient, the profession of caring and for the environment. Lausten proposed a nursing ecological theory with the goal to broaden nursing perspectives by incorporating expanded concepts of global ecosystems, communities and interrelationships derived from ecological sciences. Hereby, nurses face a challenge to incorporate environmental concerns as well as ecological beliefs into their professional duties. Ecology and sustainability in nursing care is discussed by Anåker and Elf (2014) and Kangasniemi, Kallio, and Pietilä (2014) as being a present-day issue in health care, with the goal of maintaining an environment that does not harm current and future generations’ opportunities for optimal health. The term *ecological perspective* in caring is also used and proposed in research for creating better understanding of creating optimal care for elderly institutionalized patients to identify risk factors associated with elder abuse (Lawrence et al., 2016).

Even though these promising efforts of developing ecological theory in nursing manage to place an emphasis on environmental concern and ecological beliefs, within health practices, they do not explore the meaning of *ecological care per se*, for example, how caring practice recognizes the interrelationship and interdependence between humans and our living context, as well as between humans, and what consequences these interrelationships have for a health-oriented care.

## A study of anthroposophic care

Anthroposophic care integrates theory and practice of modern medicine with nature-based treatments and a humanistic view of humans, health and care (Arman, Ranheim, Rehnsfeldt, & Wode, 2008; Kienle et al., 2013). This form of care represents an integrative system, an extension of conventional medicine incorporating a holistic approach to illness and healing. It is practiced by physicians, nurses and therapists, and provides specific treatments and therapies including medication, specific nursing methods, arts, movement and massage therapies. The entire range of all acute and chronic diseases is being treated, with a focus on children's diseases, family medicine and particularly chronic diseases necessitating long-time complex treatments as described and scheduled in research by Hamre et al. (2007) or Kienle et al. (2013). In addition to the broad-spectrum of professional health care knowledge, health care personnel in this field possess specialized medical and nursing knowledge. At present, there are more than 25 hospitals within Europe that are specialized in this form of medicine and care (Arman, Hammarqvist, & Kullberg, 2011).

Emerging from the philosophy of Rudolf Steiner, anthroposophic medicine consists of three pillars: nursing care, therapeutic treatment and medical treatment (Arman et al., 2008; Therklesson, 2005). Anthroposophic therapy treatment is individualized by a physician and could include conventional medication accompanied by any of the following complementary therapies: natural remedies (herbs, plant extracts, essential oils and natural substances), therapeutic baths, external compresses and rhythmical body oiling, artistic therapies (clay modelling, painting, music therapy and therapeutic song), therapeutic eurhythmy (movement therapy), rhythmical massage (a light-touch massage), psychological and biographical counselling and anthroposophic nursing care.

## Data and analysis

The targeted participants in the study were 16 patients diagnosed with different types of cancer who had rehabilitative care for 14 days at the clinic. The interviews that constituted our data were part of a larger study (health care staff and patients at the clinic), probing into the meaning of existential care (Arman, Alvenäng, El Madani, Hammarqvist, & Ranheim, 2013). However, present interviews are not earlier analysed and are as such not to be considered as secondary material.

Open-ended questions such as “what does care at the clinic mean to you?” were asked. The participants gave their informed consent to participate and received both oral and written information of the project. The project was approved by the regional ethics committee (2011/444/31/4).

Interview data were analysed from a phenomenological and reflective lifeworld research (RLR) approach presented by Dahlberg et al. (2008). With its ground in lifeworld theory, RLR makes it possible to research existential phenomena, that is, how aspects of human existence are lived and experienced by humans. In this way, RLR enables a reflection on the unreflected and tacit meanings of human existence. The crucial element in this search for meanings is the researchers’ openness. With a bridled attitude, one is obliged to problematize and reflect on taken-for-granted assumptions in order to let the phenomenon in question show itself more fully. The understanding process is thus slowed down in order to let new and surprising meanings arise that otherwise might have been clouded by the researcher's own pre-understandings or established meanings of the phenomenon. The bridling process is, however, not something that is performed once and for all, but rather implies an ongoing reflection throughout the process of planning the project, data gathering and analysis as well as reporting the study.

In this study, focus was on the phenomenon health-oriented care and to describe the phenomenon's meaning structure, that is, the essential and more general meanings as well as contextual nuances and more individual meanings, as they appeared in the interviews. Our analysis of the said data had the same aim as the whole study, that is, to explore the meaning of ecological care.

## Empirical findings: Lived experiences of health-oriented caring

The participants felt strongly about the importance of care that addressed them as whole beings. The anthroposophic care means “a life-line,” that is, care that relates to the patients as individuals with and within their life context. The common objection was against care that was described as fragmented and that addressed only their care in silos.

The experience of care at the anthroposophic clinic was influenced by and connected with the therapies as well as the attitude of the care-givers, all within a caring milieu including architecture, the choice of materials in the buildings, linen, cloth, choice of colours and forms, food choices and contact with nature. The practice within the caring milieu nurtures the patient and their existence and is perceived as supportive health.

The care can be described as providing opportunities for the patients to be both passively receiving care and actively engaging in their own health decision-making processes. On the one hand, they were received and accepted as patients, that is, as people with caring needs, who need health guidance. Consequently, they were given all-encompassing nursing care that addressed their vulnerabilities. Opposed to that, patients were of the view that they were viewed as people with resources and experts with regard to their own existence. They were empowered with “tools” for self-reflection and personal growth through carefully chosen treatment and other creative activities that addressed their health needs.

This approach towards health-oriented care can be viewed as ecological with regard to several aspects. Firstly, it is characterized by placing the focus on the patient, to be viewed as individuals, especially integrated in a living network with other humans, in a community as well as nature. The patients reported that they were not addressed as (someone with) an illness or a diagnosis but were met with sensitive care towards their individual patient needs, with the aim to adhere to the individual care and health challenges as well as their health regimes in relation to their own approach to life. This mounts to both a biological and an existential understanding of the patients, including therapies that unite these perspectives. The care is characterized by a high degree of responsiveness and sensitivity in order to bridge the individual patients’ needs between passively receiving care and taking responsibility for their own healing outcome. In this approach, the main focus is on health, not illness. When patients are allowed to participate in their own health and caring responsibilities, their experience of life power and life meaning is strengthened.

The phenomenon is further described by its constituents, which make the essential meanings more explicit. The constituents open up for more contextual meanings and individual descriptions from the interviewees. The constituents are: “Nursing concern, peacefulness and letting be”; “Realization of one's intentions as of letting be”; “Therapeutic treatments and cultural input”; “The meaning of the environment”; and “Green and white caring.”

## Nursing concern, peacefulness, and letting be

Existential vulnerability increases when one is diagnosed with severe illnesses or other conditions, which threatens well-being and the capacity to realise one's life goals. In these instances, care that expresses nursing concern and attention is immensely important. The patients in this study expressed how they felt that they were taken care of and protected from the threats that accompany illness.

Adjacent to this meaning was the experience of peacefulness. The clinic offers a milieu of silence, without distractions, such as traffic, intensive discussions, television or computers. Instead, the patients are offered experiences from the surrounding countryside landscape, storytelling or handcraft activities. The patients appreciated the fact that “even the care-givers speak mindfully”; they expressed that “everything here is taken care of.” This peaceful milieu supports the development of one's own inner peace.

Descriptions like the feeling of not being in a clinic but rather some kind of resting or rehabilitative home came forth. The interviewees referred to the importance being able to reach into deeper settings of rest, “there is no background noise here, as there is at other hospitals, nor any luminous tubes or noisy air vents here or doors that slammers; it becomes clear that someone has done some thinking about this.”

Expressions of the opposite were also present, giving the insight that even the experience of peacefulness is on an individual basis. Some descriptions expressed restlessness and frustration from the silenced mood that is a permeated atmosphere at the clinic: “… sometimes I just wanted to spin off—or wish for someone to get stressed, so that I could feel that the usual life conditions were present here as well.” Stillness/silence is not always experienced in a positive way. There are expressions in data that show how encountering and benefitting from stillness/silence could be a challenge.

However, the clinic makes up a kind of “caring cocoon.” The brutal reality is on hold for a while by the caring attention that the patients are wrapped in, whilst they are surrounded by a health-oriented caring environment. Even if such care, as we point to above, can be experienced as a challenge, the interviewees expressed appreciation of the nursing care, which made them feel important and safe. The caregivers, together with the caring context including other patients, invited tranquillity and provided room for patients to be “as we are.”

## Realization of one's intentions as opposed to of letting be

According to our data, the experiences of peace, stillness, rest and letting be are not opposite of being active. Having been able to let go of everyday demands and the distress that illness causes, the patients’ apt for well-being and the capacity of realizing one's intentions are experienced as being strengthened. The care seems to infuse the patients with energy and growth. The care that was in focus in the interviews was experienced as providing the patients with desirable pauses from a sometimes hectic everyday situation. The importance of rhythm in the daily routines was appreciated by the patients. They had time for reflection and were able to establish a new and healthier life rhythm, which included both movement and stillness.

The interviewees expressed how the attentive care was experienced as confirming and strengthening, giving room for accepting and affirming oneself, to see oneself in new light: “I came in contact with creative processes—and something happens in my body.” And then the possibility to change something in one's situation increases. The patients were given an opportunity to build up the strength that illness demanded from them.

In-patient care always runs the risk of forgetting that patients are individuals with the majority of their lives outside of the clinic. It is therefore interesting to note how the interviewees in this study expressed how they found support for their further existence, in particular when they would leave the clinic. The care that addressed the patients as individuals enabled them to find themselves and what they wanted to do in the future. For example, they learned strategies for how to feel what is right and what is wrong for them; they learned how to protect themselves; they found ways to say “no” to too much work or too many engagements; and they learned how to care for others, by caring for themselves. Maybe most important is the insight of the significance of that impact on their own lives. The participation in both health and caring processes during the time in care awakes one's power to take responsibility for one's health into the future.

## Therapeutic treatments and cultural inputs

There exists a variety of caring tools within anthroposophic medicine and care (see method section). Nurses, physicians and therapists work together as a team in addressing the individual patient's needs. Dwelling on the humanistic view that the human being needs the threefold of truth, good and beauty to develop and to heal, care offers a variation of cultural inputs and activities to stimulate the individual patient. There is a library with scientific literature, as well as essays and poetry at the clinic. Live music is performed and performing art and local theatre are presented. Architecture is intentionally constructed to contribute to a healing environment in terms of forms and colour.

Contributing cultural inputs or events such as storytelling evenings were expressed as both entertainment and a source for deeper reflection. The stories have the possibility of explaining life situations or essentialities of life in allegoric meanings: “Stories or fairy-tales have the ability to explain the larger questions in life.” The story telling evenings, which took place in the common living room where the patients and staff gathered to listen to, were appreciated: “I haven't heard stories told since I read them for my children.” One's own reflection on the stories could be shared with the others, or just be restrained and privately contemplated.

The patients may have achieved new insights of themselves through the works and activities done with artistic therapy that also is part of the caring concept. Important was the room for being creative without being judged or validated, letting the process itself being what mattered: “No judgments were made in the creative processes …”. Another interviewee added: “I baked my anger into the clay in modelling …”. The art therapy was an example of how care sets reflections in motion and how the patients could express themselves in new ways.

A special kind of therapeutic input is eurhythmy, which in addition to physiotherapy is an often used movement therapy. The principle of this therapy is that life processes are stimulated through certain movements. Therapeutic eurhythmy attempts at enliven and support flux in processes: “When I came I had a lot of fatigue in my body, then in the eurhythmy sessions things happened, it affects me a lot and I become thirsty and tired. Working with movements in relation to the central points in self, my experience was that I was building up stamina within myself on which I can hold on to.”

## The meaning of the environment

In anthroposophic health care, the ambiance of spaces is understood as having impact on the people using the space. The importance of including aesthetic enhancements in attempts to reduce stress and anxiety and increase well-being is a central thought behind environmental design. The consciousness of creating and providing positive contexts to become actively healthy is central. The meaning of such environment reaches the patients:

The architecture with deliberately designed shapes and forms, imagine I have beside my bed a window allowing me to view straight into the sky — quite amazing … being able to lie in bed with the nature getting in to you, the healing nature.

A central principle in anthroposophic care is the significance of warmth. Warmth mediated through the deliberate and consequent use of original materials, for instance, in the bed linen and other textiles and furniture. Warmth is the mediating factor also in treatments such as foot baths and therapeutic baths, rhythmical embrocation or as in an after lunch coating with stimulating herbs followed by rest, in warmth. The constant use of hot-water bottles or millet cushions is central. Warmth is even mediated in the deliberate and consequent use of form and colour in the wards.

The concept is the entirety of the healing milieu as it is experienced through the purity of the food, the moments of rest, the careful wrappings, the massages and embrocation, the colours and forms in the buildings and the genuine warmth. It is no coincidence that the windows are placed where they are or that the window recesses are angled the way they are or that the colours feel like being the necessary ones. The thought of a healthy existence is everywhere, according to the interviewees.

## Green and white caring

Finally, we want to describe some meanings which originate from the patients’ comparisons of the anthroposophic care with general care. In some interviews, there were expressions of the “green care” and the “white care,” where the green care was understood as human and holistic: “The green care strengthens humans and cure on a more long term basis. The white care is fine, but above all—it does not see to the whole, even if it has become better in this respect.”

Previously, I have been through a whole range of treatments and become very crumpled up. // Thee white care should ask for help from the green care … when it is about helping people to be brought to order again after having been crumpled up.

In green caring, the caring focus was understood to be, not on the illness *per se*, but on the individuals, their health and healing. Consequently, there are no quick fix thoughts of, for example, giving a swift medication only to take away pain. Rather, the concept is that of a more slow edifying process to strengthen and regenerate, to appeal to the body's own inner-strength and re-creative forces. This was appreciated by patients, whose voices may end the description of findings:

Feels like I have been helped with finding back the powers of my body and to build it up again—this is where I find conventional hospital care couldn't help me … and this is what this clinic has helped me out with.

In conventional care, there is no awareness of how important every encounter is, and one is so terribly vulnerable in one's illness.

The white care claims that we are medically ready-made when the cancer cells are gone … but they don't realize that there is a whole lot to work through in the aftermath of the tough medical and surgical treatment.

That someone really cares for you, that is not something one experiences in the conventional care.

## Excursive interpretation

The experience of (serious) illness changes the perception of oneself and one's body as well as of the surrounding world. Indeed, illness can even be defined as a loss of homelikeness in the body and in the familiar world (Rosberg, 2000; Svenaeus, 2000). When one's daily way of living is thus disrupted, one's body has stopped functioning in the usual way, and the world has become a strange place, a special form of self-awareness is needed. Then, one needs to find out both *who I am*, in this new situation, *where I am*, in relationship to other people and the surrounding world, and *where I can go next*, or in other words, what my possibilities or ways of confronting this new situation are. The crucial task of ecological care is to help the patient to once again find one's place in a world that is characterized by interconnectedness, something which is expressed in our data as becoming “centred” or “balanced.” From this “centred” place, it is possible to act and to find new ways to health. Ecological care is not only about fighting an illness, but also recognizes a patient from inside a world that s/he is affected by and affects, that s/he is understood and understands from. Such care tries to restore this connection by making possible the rhythmical movement as well as the space in-between activity and rest, between being cared for and actively involve oneself in one's recovery and between closing oneself off from the world and once again going out into it.

From the empirical exploration, we learned about how patients express their appreciation of *both* a nursing concern that leads to peacefulness and letting be *and* care that allows for their own life power to act in a way that strengthens their existential intentions and supports realization of them. In such environment, it is not about either–or, but both, that is, both being able to rest and getting tools to actively engage oneself in life. Receiving care that is characterized by nursing concern could spontaneously be understood as care that fosters passive patients. What we found, however, was that care, which includes peacefulness, rest, stillness and silence, makes room for activity. For the patients in our study, there is a steady movement between passively receiving care and being active in their health processes. It is also the opposite: activity supports the experience of rest and recovery. Instead of getting stuck with the old dilemma of either–or, we would therefore now like, with the help of the philosophy of Merleau-Ponty, to launch an idea of ecological care that explains how passivity can be the other side of activity, or in other words, that recognizes the rhythms of existence.

Merleau-Ponty's philosophy (1995/1945) displays an ontological understanding of human existence that is characterized by resistance against contemporary dualism, for example, between body and soul, materialism and idealism, external and internal, or movement and rest. Instead of seeing these aspects of life as radically separated categories, he explains how they belong together and define each other, as figure and background. Seeing these mentioned and other dualities as figures and backgrounds means that we outline areas of in-between, areas that do not belong to one of the two sides but are created by the interaction between them, for example, between thinking and feeling, persons and their environment, or myself and the other.

One way of understanding the duality (but not the dualism) of existence is through rhythm. Merleau-Ponty emphasizes the rhythms of existence that belong to and characterize our existence because we are bodily. Thus, we go in and out of sleep as well as in and out of relationships with other people and with the world. Even illness can be seen as a rhythm of existence that we move into and out from. If we take sleep as an example, sleep is never absolute; it is the other side of wakefulness. Wakefulness thus always remains as a background and possibility for our sleeping state (Merleau-Ponty 1995/1945). In the same way, illness is not absolute. Even if it interrupts our everyday world as well as the health that we had taken for granted, and places us in another world, one where we perhaps do not recognize ourselves and do not feel at home, our everyday (healthy) world will remain as a background for illness, as a possibility that is always present. “[T]he sleeper is never completely isolated within himself, never totally a sleeper, and the patient is never totally cut off from the intersubjective world, never totally ill” (Merleau-Ponty, 1995/1945, p. 164).

To understand this rhythmical movement of existence is to understand how it contains both active participation in the world and withdrawal from it, that is, both movement and rest. Our existence has the power of directing us towards others, towards the living present and the future, but it also has the power of arresting this existential move, of shutting us up, of being “the place where life hides away” (Merleau-Ponty, 1995/1945, p. 164). All this is because we are bodily, because it is, as Merleau-Ponty phrases it, the body that sustains “the dual existential action of systole and diastole.”

At the very moment when I live in the world, when I am given over to my plans, my occupations, my friends, my memories, I can close my eyes, lie down, listen to the blood pulsating in my ears, lose myself in some pleasure or pain, and shut myself up in this anonymous life which subtends my personal one. But precisely because my body can shut itself off from the world, it is also what opens me out upon the world and places me in a situation there. The momentum of existence towards others, towards the future, towards the world can be restored as a river unfreezes. (Merleau-Ponty, 1995/1945, p. 164)

Following Merleau-Ponty, one of the tasks of the professional caregiver, and an essential component of ecological care, is to restore the patient's connection with the everyday world, to once again make possible the momentum of existence and, in this way, to create the opportunities for the river to unfreeze. Or, in other words, to once again find the everyday, healthy world, not on the other side of the world with illness, but inside and around it, relating to each other as figure and background.

Understanding the rhythms of existence and their importance for health means to bring an element of everyday life into the world of the ill patient, even in such subtle examples as seeing nature through the window of the hospital bedroom. It also means to awaken the patient's life power and life meaning by the use of an initial constraining action, by creating bounds for the patient, such as is done in the anthroposophic nursing care in, for example, foot baths and rhythmical embrocation. Such care and touch can be thought of as a way to constrain the world of the patient. But as our analysis shows, as well as a study by Ozolins (2011), it is rather the opposite that happens. To become aware of one's own boundaries through an attentive carer's touch makes room for acceptance and affirmation—for *letting be —*which, in its turn, makes it possible to look at oneself in a new light, and to see possibilities for change. This existential duality, or in other words, this rhythmical caring, where a limitation in fact opens up new possibilities can be understood from Merleau-Ponty's ontology, as with movement that is not the opposite but the other side of rest, where constraint is the other side of open possibilities.

The importance of finding rhythm in existence showed itself in our findings as the active engagement of one's life being nothing but the other side of the experience of peacefulness. The impulse to once again become active in one's life and health processes is born from rest and peace provided by the nurses’ thoughtful care. To once again find one's place in a changed world and in a changed body is equally made possible by this kind of care. Therapeutic treatments and cultural inputs are ways of helping the patients finding their own place in a vulnerable situation and in the world by the means of, for example, stories or allegories that gives perspectives on one's own situation and places it in a bigger context. Certain movement therapy, for example, in the form of eurhythmy is a way of finding central points and balance in one's body as well as how one's body relates to the physical surroundings. The significance of warmth and the feeling of being “wrapped in” can equally be understood in terms of rhythm and place, firstly in the way that it provides rest, and from rest, a possibility for new movement. Secondly, warmth and wrapping in the form of, for example, rhythmical embrocation can also be seen as a way of finding one's place in relationship to the world and other people.

To experience a carer's warm hands in rhythmical embrocation is a way to experience one's own contour, one's own border. Merleau-Ponty (1995/1945, cf. Dahlberg, 2013) explains how our boundaries as human beings are not clearly and once and for all defined, but are constantly being drawn anew in our engagement with things and other human beings. It is in this relationship of connection and at the same time separation that we find and develop our contours. The meeting between the patient's (lived) body and the carer's physical touch provides the possibility for the patient to become aware of and know her body. It makes the patient's body more present and more explicit. The two bodies, for example, the carer's hand on the patient's body, constitute each other's borders and thus create a new room between them. Between these two bodies, there is a common space opening up, a space that they share and that at the same time defines the border between them, their contours. It is an in-between space that also can offer a moment of rest, of just *being who I am*. The patient, who is otherwise moving, engaging in the world, can come to a momentary rest, can rest with himself/herself and discover himself/herself.

It is, however, important to note that we are not talking about a complete standstill, about a once-and-for-all definition (e.g., a diagnosis would be). The meeting between the carer's hand and the patient's body instead offers a momentary pause, a possibility for self-reflection, a *space* in which the patient can discover himself/herself and his/her possibilities. This in-between space can also open up new possibilities, such as *this is who I could be* (Dahlberg, 2013). It can be thought of as a centre point from which to move.

The importance of finding one's place in a world of interdependent relations can also be seen in the last two constituents, the meaning of the environment and green and white caring. Besides providing peace, a thought-through healing milieu helps the patients finding their place in relation to their context. The accessibility of the surrounding nature through, for example, the placement of buildings and windows can either support or hinder this process. As the patients in the interviews expressed, illness does not only reside in the cancer cells but changes a person's entire situation and understanding of himself/herself and his/her body. Accordingly, health is not solely the removal of cancer cells. Ecological care, here expressed as “green caring,” is a way of addressing “all the rest,” that is, of again finding one's place in a new situation and in a changed body and world.

## Conclusive reflections

Ecological care recognizes that human existence must be understood within a web of relationships. This is not to say, however, that every individual's place in the world is self-evident or self-given (as is often taken for granted in several approaches of “holistic caring”). On the contrary, one continually understands oneself anew in relationship to one's surroundings, and the surroundings are perceived anew in relation to oneself. Becoming a patient means that both the self and one's world change, and the task of ecological care is not only to see the patient within a world of relationships, but to help the patient *find his/her place again, to understand himself/herself and the world anew*. Ecological care thus promotes rest and self-discovery, and from this “passivity,” a new form of “activity.” Such care recognizes how the everyday, healthy world is not completely gone when suffering from illness, but there to be discovered anew.

As we can see from our analysis as well as from other studies (cf. Arman et al., 2008; Fjelland & Gjengedal, 2008; Norlyk, Martinsen, Hall & Haahr, 2016; Van Wijngaarden, Leget & Goossensen, 2015, 2016), patients have a desire to be seen and recognized as individuals in their personal context. They certainly do not want to be reduced to diagnoses, symptoms or to other parts of their being, but they need tools to address the disconnectedness caused by illness (cf. Van Wijngaarden et al., 2016) or by treatment (Norlyk et al., 2016). Patients need care that addresses them as persons that belong to, but are not reducible to, a world characterized by social, cultural and natural relationships (cf. Nilsson, 2009). They need care that can relate to their suffering but also to their possibilities of changing the relationships that they are part of, that is, to their own on-going and changing efforts to live as fully as possible, even despite illness. Such care that patients ask for may include treatments inspired by the anthroposophic care, which meet basic human needs, such as therapeutic baths, external compresses, and rhythmical body oiling and massage, artistic therapies, for example, music therapy and movement therapy.

Our findings illustrate how ecological care means a combination of medically and biologically oriented treatments, with existentially oriented care. Such care supports patients recovering from illness or in learning how to live with illness (cf. Berglund, 2014). Patients want to be acknowledged both in their biology and with their thoughts and plans for their life as well as with their emotions and hunger for what is meaningful.

Individuals who become patients need ecological care that does not deny any aspect of what it means to be a patient. First of all, patients must be seen in their existential vulnerability. Being ill and in need in of professional care means that their autonomy and strength to take active charge of their illness and life situation is momentarily undermined. On the other hand, they must be acknowledged also in their freedom and capacity to strive towards a greater experience of health, that is, experiences of well-being together with the capacity of living forward according to their own meaningfulness. Freedom is but the other side of vulnerability. Being in a vulnerable position where one's hitherto successful strategies of living and finding meaning is questioned is at the same time is a possibility for finding new ways of approaching existence. Ecological care recognizes this, and instead of demanding from patients’ strength and autonomy that they at present do not have, it seeks to promote such strength and autonomy. Ecological care helps patients back into the rhythms of existence.
